# Authors’ Reply: “Evaluating GPT-4’s Cognitive Functions Through the Bloom Taxonomy: Insights and Clarifications”

**DOI:** 10.2196/57778

**Published:** 2024-04-16

**Authors:** Anne Herrmann-Werner, Teresa Festl-Wietek, Friederike Holderried, Lea Herschbach, Jan Griewatz, Ken Masters, Stephan Zipfel, Moritz Mahling

**Affiliations:** 1 Tübingen Institute for Medical Education Faculty of Medicine University of Tübingen Tübingen Germany; 2 Department of Psychosomatic Medicine and Psychotherapy University Hospital Tübingen Tübingen Germany; 3 University Department of Anesthesiology and Intensive Care Medicine University Hospital Tübingen Tübingen Germany; 4 Medical Education and Informatics Department College of Medicine and Health Sciences Sultan Qaboos University Muscat Oman; 5 Department of Diabetology, Endocrinology, Nephrology Section of Nephrology and Hypertension University Hospital Tübingen Tübingen Germany

**Keywords:** answer, artificial intelligence, assessment, Bloom’s taxonomy, ChatGPT, classification, error, exam, examination, generative, GPT-4, Generative Pre-trained Transformer 4, language model, learning outcome, LLM, MCQ, medical education, medical exam, multiple-choice question, natural language processing, NLP, psychosomatic, question, response, taxonomy

We appreciate the thoughtful commentary titled “Evaluating GPT-4’s Cognitive Functions Through the Bloom Taxonomy: Insights and Clarifications” [1] and welcome the opportunity to clarify and expand upon our research findings [2] regarding GPT-4’s cognitive evaluation using the Bloom taxonomy.

First, we acknowledge the confusion surrounding the use of the term “difficulty” in our manuscript. Traditionally in educational testing, “difficulty” is quantified by the ratio of correct responses against the number of students taking the test [3]; thus, a rating of 1 indicates an extremely simple question (100% correct responses), and a rating of 0 indicates a significantly challenging question (0% correct responses). Throughout the manuscript, we used “difficulty” as a measurement scale.

Consequently, “higher difficulty” means it is higher on the scale and thus easier. This also applies to Figure 3. Because “lower” means less easy (ie, closer to 0 on the scale from 0 to 1), it shows that the questions answered correctly were easier compared to those answered wrong. Although our use of the measurement “difficulty” is correct, on reflection, we agree that we could have been clearer, and we apologize for any confusion.

Second, the commentary on GPT-4’s approach to “memory” tasks adds a valuable dimension to our discussion. We agree that GPT-4 “remembers” through technical and programmatic means, highlighting the critical difference between GPT-4’s architecture and human cognitive processes, a distinction that was central to our study.

However, GPT-4’s material selection is far more complex than a flat-file database with simple mapping (unless the exam questions had been in the testing data, but this is not applicable in our case). Generative tools like GPT-4 have other weaknesses and strengths. For example, they may perform relatively poorly on pure memory-recall problems but excel in topics requiring subtlety and nuanced work. This is demonstrated by GPT-4’s high performance on soft-skill questions from the USMLE (United States Medical Licensing Examination) and AMBOSS [4]. Part of our study went further by using the Bloom taxonomy as a framework for tracing the logical process of GPT-4’s *explanations* (not *answers*) and determining the stages at which its errors occurred.

This discussion underscores a critical point: the complexity of assessing artificial intelligence and the processes underlying the output of models like GPT-4. This methodology allows us to critically examine where GPT-4’s responses fall within a spectrum of cognitive tasks, from simple recall to more complex analytical and evaluative processes.

Third, while it is quite true that many questions in medical qualifying exams are simple memory-type questions, we see this as a weakness rather than an optimum aiming point. While our understanding is that medical schools are trying to move away from those types of questions, this is an area of further research.

Again, we thank the author for the thoughtful critique of our paper and the resultant continued discussion, which underscores the importance of ongoing dialogue and research into artificial intelligence’s cognitive processes and how they parallel and diverge from human cognition.

## Abbreviations

USMLE: United States Medical Licensing Examination
